# Medical Model in Caries Management

**DOI:** 10.3390/dj7020037

**Published:** 2019-04-01

**Authors:** Madeline Jun Yu Yon, Sherry Shiqian Gao, Kitty Jieyi Chen, Duangporn Duangthip, Edward Chin Man Lo, Chun Hung Chu

**Affiliations:** Faculty of Dentistry, The University of Hong Kong, Hong Kong, China; yonjunyu@hku.hk (M.J.Y.Y.); gao1204@hku.hk (S.S.G.); kjychen@hku.hk (K.J.C.); dduang@hku.hk (D.D.); hrdplcm@hku.hk (E.C.M.L.)

**Keywords:** medical model, caries management, prevention, caries risk, remineralisation

## Abstract

The current mode of dental caries management mainly operates through irreversible and symptomatic treatment by means of drilling and filling, while caries prevention is largely overlooked or omitted. Focus should be redirected through a medical model towards elimination of the disease through tackling its causes and risk factors to address current and future caries initiation. Caries is the demineralisation of dental hard tissues by bacterial acids when periodically exposed to fermentable carbohydrates. The medical model of caries management is a philosophy that steers sustainable caries management through controlling bacterial infection, a reduction of risk levels, remineralisation of teeth and long-term follow-up. Its goal is to prevent new and recurrent caries, arrest ongoing caries processes by alteration of the cariogenic environment, and support the healing of remineralisable enamel and dentine. The mechanism involves dietary counselling and plaque control, placement of dental sealants, administration of fluoride agents and chemotherapeutic medications and use of chewing gum. This paradigm shift from a surgical to a medical model aims to pursue the ultimate intention of maintaining a caries-free dentition and is anticipated to promote true oral health-related quality of life. The objective of this paper is to discuss the medical model of caries management.

## 1. Introduction

Caries remains a prevalent disease worldwide, affecting every region, regardless of age and socioeconomic status. In the Global Burden of Disease study, untreated caries in the permanent dentition was the most prevalent disease of humankind in the past decade, and untreated caries in the deciduous dentition was ranked the tenth. Despite advancements in dental technologies, caries prevalence and incidence have remained largely the same [1]. Is the current direction of dental health maintenance misdirected and the root causes and risk factors of caries not properly addressed?

Caries is associated with pain and mortality because of the disease itself and even more so, the treatment of caries. It is one major cause of tooth loss or extraction in all age groups, particularly in the first few decades of life [2,3,4]. A substantial portion of dentists’ time and effort have been placed on treating cavities by restorative means, including with new or replacement fillings [5]. However, treating *cavities*, which are signs, is not quite the same as treating *caries*, the disease. It is quite certain now within the dental scientific community that simply the removal of so-called “gangrenous tissue”, as it was referred to in the past, or “diseased structures” of the tooth, is no longer the contemporary approach given the ability of dental hard tissues to heal following appropriate interventions, such as remineralisation therapy [6]. Caries is gradually being regarded and recognised as a disease in the oral cavity involving net demineralisation of the dental hard tissues that occurs as a result of pH imbalance or acidity-facilitated cariogenic bacteria in the presence of fermentable sugars [6]. The FDI World Dental Federation further classifies caries as a ‘non-communicable’ and ‘behavioural’ disease to emphasise the behavioural aspects of the disease [7].

Caries management used to be a significant part of the dental business, both in the practice model and in terms of income proportion [8]. Patients present with different severities and magnitudes of the caries disease every day that require management strategies of different complexities. An array of treatment options is available, from the simplest topical fluoride to the most complex reconstruction to recreate an entire tooth crown that is destroyed by caries. Clinical judgement made by the dental practitioner determines the combination of strategies to be used, but it is the philosophical model behind caries management that dictates this. Broadly speaking, major caries management approaches can be classified as following one of two models: the medical model and surgical model.

The medical model for caries management is a philosophy where optimising dental health by eliminating caries is the primary aim. Under this model, the onset of caries disease should be prevented, ongoing current disease processes should be arrested and eliminated, and damage should be reversed as far as possible. On the other hand, the surgical model involves the cutting and drilling of teeth with cavities and replacing them with artificial materials to restore the tooth to form and function. Both models have their strengths and value; however, when the focus is on elimination of the caries disease, it is apparent that the medical approach to the caries disease should prevail.

## 2. Caries Management Approach and Medical Model

In the context of caries management, the aim of the medical model is to improve and maintain a person’s dental health by preventing caries from occurring, stopping caries from progressing and reversing damages caused by caries. The surgical model of caries management, however, focuses on treating signs and symptoms, i.e., preparing cavities and rebuilding the tooth back to its original form and size. With a better understanding of the caries disease by dental professionals, cavities are clearly not the cause of caries but are a sign or manifestation of the disease. Without dealing with the actual disease process or aetiology, the surgical model does not really address the cause and process of caries; caries has not been cured, and the disease is expected to continue to propagate. Further differences between the medical model and surgical model can be illustrated from three perspectives: caries prevention, caries arrest and reversal of damages.

‘Prevention is better than cure’—the medical model of caries management addresses the preventive aspect of the caries disease. Healthy dental tissues are shielded against caries by fluoride application, fissure sealants and dietary alterations or improvements. Even though the surgical model claims to be of a preventive nature by cutting away ‘caries-prone fissures’, this does not avoid new caries from initiating on a newly cut surface. On the contrary, the surgical model weakens the tooth structure because more natural tooth substance is cut away.

When caries has already occurred, the philosophy of the medical model lies within stopping the caries process and tackling the disease directly. The cause of caries is an interplay between a frequent intake of fermentable sugars and an ecology of cariogenic bacteria on tooth surfaces which is further aggravated by the lack of protective factors such as saliva. Under this model, caries should be treated by restricting frequent sugar consumption through dietary counselling, establishing proper oral hygiene to remove bulk bacterial load and the cariogenic plaque they produce, and chemotherapeutic methods that selectively reduce the caries-causing bacteria. Cavities may be selectively filled if this helps maintain a cleansable and cariostatic environment that is amenable to oral hygiene. In fact, complex restorative treatment and replacements are irreversible procedures and should be reserved only in cases where function and aesthetics are already compromised and more advanced procedures are required to benefit the patient through the use of contemporary and scientifically proven clinical techniques and materials.

If, however, caries is approached in a surgical manner, cavities are merely *filled*, and nothing is done to actually *restore* the caries balance. It does not stop the ‘disease activity or progression’, the absence of which is a requirement in the definition of ‘oral health’ [9]. Caries can still form where dental tissues are exposed, most notoriously around existing restorative margins, if the cariogenic environment is left unmodified. Even after successful restorative procedures, secondary caries is the number one cause of their failures [10,11,12,13]. Patching around dental cavities as they form results in increasingly heavily restored dentition until there is no more tooth to restore before its life expectancy is reached, which is what is known as the ‘restorative cycle’ [14]. Furthermore, remineralisable tissues, such as demineralised enamel and dentine, would be removed in the surgical process against the supposed intention to preserve natural tissues with healing potential. Under the medical model, remineralising agents such as topical fluorides should be applied and as long as a non-cariogenic microenvironment is maintained, caries-affected dental tissues could heal.

This paradigm shift falls in line with the objective of the popular minimally intervention dentistry (MID) concept to retain more healthy and functional teeth into their old age, especially in a world with a global ageing population [7]. The sustainability of the medical approach justifies the transition to the medical model for caries management. Caries is addressed directly from its risk factors and by enhancing protective factors, providing what the surgical model of caries management has not been able to achieve. Remineralisable tissues are supported for healing with remineralising agents. Furthermore, the patient is placed on long-term review for future prevention and early detection in the case of new disease. Overall, there is a reduction in pain and dental anxiety as a consequence of caries and its treatment. On the contrary, the traditional surgical model where caries management only focuses on symptomatic treatment and the removal of carious tissues to receive artificial tooth tissue replacement could probably be regarded as being outdated and short-sighted at best and is destructive to the longevity of the human dentition. Outcomes for caries management should be re-evaluated and hence the medical model should supersede the surgical model as the ‘golden standard’ for eliminating the caries disease. In a broader sense, eliminating the caries *disease* could be attainable with the medical model philosophy, in addition to treating disease signs and symptoms.

## 3. Medical Model in Caries Management

In consideration of the above, four essential components are to be included as part of the medical model if the management of caries is to be successful: (1) control of bacterial infection; (2) reduction of risk levels; (3) remineralisation of teeth; and (4) long-term follow-up. Although these components may be discussed separately in scientific literature, their connection and relationship in a medical model has not been thoroughly considered.

### 3.1. Control of Bacterial Infection

Caries results from acid production by acidogenic bacteria that forms and thrives in plaque biofilm in the presence of fermentable sugar. The relationship between caries and the bacteria responsible for the disease has been studied in the past. Recently, the role of bacteria and the aetiology of caries has been explained by the ecological plaque hypothesis, which encompasses ideas from the previous specific and non-specific plaque hypothesis as the cause of caries. The ecological plaque hypothesis is understood where specific pathogens which are commonly referred to as the ‘cariogenic bacteria’ are normally present in all tooth sites, even sound tooth tissue surfaces, and caries disease occurs as a result of an imbalance and environmental change in the biofilm that shifts the ecology into favouring the growth and metabolism of these ‘specific’ pathogens, most notably, *Streptococcus mutans* and lactobacilli. These pathogens are able to endure and create acids which lead to the formation of cavities, or so we call it, ‘caries’ [15].

In response to caries as an infectious disease, chemotherapeutic medications targeting bacterial biofilms would reasonably be expected to control the disease [16]. A number of antimicrobials have been marketed and their effectiveness varies substantially. Chlorhexidine (CHX) is an antimicrobial suggested for anti-caries use, but research conclusions have been inconsistent. A 2015 Cochrane review on CHX treatments concluded that the evidence for CHX varnishes or gels to prevent caries or reduce *S. mutans* count is weak [17], while the same antimicrobials appear to inhibit caries of up to 46% according to a meta-analysis published in 1996 [18]. Another report suggested that data might be ‘promising’ for CHX varnishes and gels, but not CHX rinses [19]. Fluoride itself is also an antimicrobial, but its role in hard tissue remineralisation appears to be far more pronounced and clinically relevant than its antimicrobial effects at lower concentrations [20]. Xylitol, a sugar substitute, is a known antimicrobial and has been shown to interrupt *S. mutans* metabolism and reduce plaque acid formation [21]. Other topical antimicrobials such as vancomycin have been suggested for removing specific cariogenic bacteria, but the idea has been met with resistance in the view that the superfluous use of antibiotics in the oral environment gives rise to possible bacterial resistance in the host [16].

Indeed, controlling a biofilm disease would not simply be successful by dealing with only one or two ‘pathogenic’ species at a time, or using a one-drug-kills-all approach that wipes out the entire biofilm altogether considering that salivary pellicle and bacterial plaque forms all the time and is ubiquitous in the oral cavity. Furthermore, even when chemotherapeutic methods have been shown to reduce microbial counts, this might not directly translate into caries reduction [16]. In spite of this, chemotherapeutic medications such as topical chlorhexidine may be used to some success to target the microbial aetiology of caries in individuals with high cariogenic bacteria counts.

### 3.2. Reduction of Risk Levels

An individualised caries preventive plan may be formulated for patients who have undergone proper caries risk assessment [22]. The sequence first involves an identification of caries risk factors by the clinician, followed by calculation according to the significance of each risk factor towards future caries incidence. The third step is used to produce a preventive plan that may be unique and appropriate to the patient’s clinical conditions and sociodemographic background in order to tackle the caries disease in the most effective and practical manner.

Numerous risk factors have been reported throughout scientific literature that contribute to varying extents with the initiation and propagation of the caries process [22]. Examples of caries risk factors explored involve clinical (past caries experience, time since tooth eruption, tooth site and morphology, denture or appliance usage), plaque or oral hygiene level, biochemical (saliva quality and quantity due to radiation or drug therapy, salivary proteins), microbiological profile, socioeconomic (education or family background, social level, income) and lifestyle parameters (diet, nursing and oral hygiene habits, history of drug abuse). The significance of each type of risk factor (in terms of odds ratio or relative risk) and accuracy of caries risk level prediction using single or a combination of risk factors (in terms of sensitivity and specificity) have been tested in many populations [22]. Existence or co-existence of multiple factors in the same individual informs about his or her heightened caries risk and necessitates additional risk reduction measures.

Caries risks, however, are not static, but change throughout the life of an individual. Factors significantly increasing a child’s probability of having caries may play a lesser role in caries progression to the same individual as an adult. Elderly persons, on the other hand, are distinctly influenced by risk factors such as xerostomia and gingival recession by increased exposure of root surfaces [19]. It could be safely inferred that even risk management strategies differ across the age group, which further accentuates the importance of individualised risk assessment and tailored management plans.

The caries preventive plan incorporates risk reduction strategies from multiple perspectives in order to be effective. It involves the input of professional advice and treatment, as well as patient input by behavioural or lifestyle modifications. From the point-of-view of practicality and efficacy, caries risk reduction is an integration of four approaches: (1) dietary analysis and advice to reduce sugar intake; (2) plaque control by reinforcing oral hygiene; (3) use of chewing gum to increase salivary protection; (4) dental sealants to prevent fissure caries; and (5) use of fluoride. The use of fluoride pertains to the remineralisation of dental hard tissues, which is a major component of the medical model in caries management, and is elaborated in detail in the next section.

Frequent sugar intake is a major cause of dental decay and is additionally responsible for other health threats, most notably, diabetes mellitus. The presence of simple sugars in the oral cavity perpetuates the caries process, favouring certain acidogenic bacteria and the formation of cariogenic plaque. The frequency to which an individual is exposed to fermentable sugars, including sucrose or its monosaccharides, greatly accelerates the formation of caries [23]. Reducing the frequency of intake of cariogenic carbohydrates lowers an individual’s caries risk. Recommending lower daily sugar consumption by means of a dietary consultation and advice to consume less cariogenic alternatives is most often the suggested method to cut down on fermentable carbohydrates and discontinue the caries process.

Plaque control by the individual is intended for disrupting the cariogenic biofilm that is favourable for cariogenesis. Oral hygiene instruction by a dental professional, such as a dentist or dental hygienist, is essential in reinforcing the use of a correct technique for plaque removal by the individual on a daily basis. The effectiveness of plaque control lies not only within the removal of plaque, but also with the concurrent use of fluoridated dentifrice. Proper plaque control is a prerequisite for teeth without decay or periodontal disease.

While destructive factors such as sugar and cariogenic biofilms are taken care of, protective factors should be enhanced to further minimise caries risk as far as possible. This includes increasing salivary protection, use of fluorides for remineralisation of dental hard tissues and fissure sealants.

Saliva stimulation utilises saliva as a natural protection against caries by its flushing activity; reservoir of calcium, phosphate and fluoride ions; and buffering potential against a drop in pH [21]. It also contains proteins with antimicrobial and anticaries activity [24]. Reduction in salivary content, regardless of cause, has been shown to increase the risk and rate of cariogenesis. Use of chewing gums, pastille or lozenges that are sugar free or with sugar substitutes (such as xylitol or sorbitol) stimulates salivary flow and is protective against caries and may be recommended for individuals [25,26].

Finally, dental sealants remain a valuable armamentarium in the fight against dental caries. Systematic reviews have shown that pit and fissure sealants are effective in reducing caries incidence with similar outcomes between resin and glass ionomer varieties [27,28]. Glass ionomer sealants might possibly have an additional cariostatic benefit by local fluoride release. Sealants reduce caries risk by mechanically blocking the accumulation and formation of cariogenic plaque from uncleansable deep pits and fissures and alter the microenvironment within the fissure into a cariostatic one. A recent guideline has reflected the strength of the recommendation of fissure sealant placement in tooth surfaces at risk of caries [29].

### 3.3. Remineralisation

Remineralisation of enamel and dentine damaged by the caries process is an essential step in promoting the healing of these tissues and protecting them from future cariogenesis to maintain their integrity. A great diversity of products containing remineralisation agents have been marketed, which may contain different levels of fluoride, casein phosphopeptide-amorphous calcium phosphate (CPP-ACP), arginine, functional tricalcium phosphate etc. Amongst the group, the chemical most extensively and successfully used for this purpose is fluoride. Fluoride remineralisation is capable of arresting the disease and even reversing the early signs of caries by the formation of an ion reservoir in plaque or on the surface of enamel in various forms of calcium fluorides [30]. Fluoride is released at different rates in various acidity levels, depending on the compound type and formulation [31], which is then available for biomineralisation. Ensuring a low fluoride level in saliva at a slightly acidic range of pH 4.5 to 5.5 maintains the formation of a fluorapatite or fluorohydroxyapatite during the simultaneous dissolution of a more soluble hydroxyapatite, thus preventing a net loss of minerals from the tooth surface [30] while increasing the acid resistance of enamel and propensity for further caries attack. These mechanisms moderate the caries process such that not only is net demineralisation prevented, but demineralised lesions may also have the potential to be remineralised and ‘healed’.

A plethora of fluoride agents have existed on the market for individual or home-use, as well as for professional application. Fluoride levels are normally measured by fluoride ion concentration in parts per million (ppm) in order to compare different forms of fluoride. The actual bioavailability of the fluoride ions determines the effect of the dentifrice in question. Currently, fluorides could be incorporated as remineralisation agents in the forms of sodium fluoride, stannous fluoride, sodium monofluorophosphate, acidulated phosphate fluoride and silver diamine fluoride. The type of fluoride compound determines its physical properties, including the solubility and stability in solution, which dictates how much of it that could be mixed into home or professional use agents. 

At the individual level, fluoride agents can be applied at the individual level on a daily basis by the use of over-the-counter fluoride toothpastes at a 1000–1500 ppm fluoride concentration following the FDI policy statement revised in 2018 [32]. Low-dose toothpastes (500 ppm or below) are also available, although guidelines for usage have varied. Taking into account the risk of fluorosis associated with fluoride dentifrice usage by young children, the most recent Cochrane review recommends dental practitioners to ‘[balance] against the risk of fluorosis’ when deciding to prescribe fluoride toothpastes [33], while other reviews have suggested simply reducing the amount of toothpastes containing moderate or high fluoride concentrations for a maximum benefit [34], or for young children to use such toothpastes (1000–1500 ppm) only when supervised, as stated in the FDI policy statement in 2018 [35]. High fluoride toothpastes (up to 5000 ppm) could be prescribed by the dentist for high risk individuals. Fluoride mouth wash are available at even lower fluoride concentrations with 0.02% acidulated phosphate fluoride or neutral 0.05% sodium fluoride. At the other end of the spectrum, dental professionals may deliver topical fluorides at a higher range of concentrations, typically from 1.23% sodium fluoride gel or foam to commonly used 5% sodium fluoride varnish (22,600 ppm) and even 38% silver diamine fluoride solution (44,800 ppm). The form of delivery is up to the discretion and preference of the dentist after the caries risk level of the patient is taken into consideration. Evidently, there is no lack of fluoride products on the market or available for use by the dental professional for the remineralisation of teeth. Remineralisation methods are versatile and the value of topical fluoride application in preventing caries and remineralising early caries lesions should never be denied or underestimated as the literature has shown it to be effective, plus it is simple to apply, painless, and low cost, compared with any other surgical methods of caries intervention.

### 3.4. Long Term Follow-Up

Successful dental health maintenance relies on long term follow-up. The follow-up has two main aims: first, to re-evaluate the outcomes of the caries intervention and plan for new interventions, if necessary; and second, to screen for new early caries lesions so that the disease can still be managed in its reversible stage. Re-evaluating the outcomes may include several aspects, including reviewing the compliance of the patient on his or her oral hygiene and dietary adjustments and checking to see if early caries lesions are arrested. At this stage, a follow-up allows the dental professional to feedback to the patient what has been achieved since the past appointment and adjust the oral hygiene and dietary suggestions. Communication between the dentist and the patient may reveal challenges to caries control and can be dealt with during the follow-up session. When indicated, topical fluoride or sealants may be re-applied during this maintenance phase of treatment. In the long run, yearly follow-up or review appointments serve to detect new changes in risk factors or behaviours, as well as screening for new initial caries so that the dental professional and patient may act towards keeping caries activity to a minimum. All this effort recalls the primary objective of the medical model in caries management, which is to optimise dental health, prevent new disease and arrest ongoing disease process. It goes without saying that without an effective recall system, the effect of successful intervention could gradually wane off until the patient returns to or reaches a state of caries imbalance again.

## 4. Conclusions

The medical model in summary highlights what is essential in order for caries management to be successful—prevention, timely intervention, support for natural dental tissue healing by remineralisation and regular maintenance—as a sustainable dental healthcare approach. The philosophy guides dental practitioners to overcome the caries disease as a result of several modifiable factors, particularly cariogenic diet and poor oral hygiene, in lieu of the pretence that caries is merely a manifestation of cavities in teeth that require drilling and filling. Controlling bacterial infection, reducing caries risks, remineralisation strategies and continued follow-up are the four necessary components of the medical model. The paradigm shift from a surgical to a medical approach would promote the longevity of a caries-free dentition of an individual.
